# Hybrid intraoperative imaging techniques in radioguided surgery: present clinical applications and future outlook

**DOI:** 10.1007/s40336-017-0235-x

**Published:** 2017-06-27

**Authors:** S. L. Bugby, J. E. Lees, A. C. Perkins

**Affiliations:** 1 Space Research Centre, Michael Atiyah Building, University of Leicester, Leicester, LE1 7RH UK; 2 Radiological Sciences, Division of Clinical Neuroscience, School of Medical, University of Nottingham, Nottingham, NG7 2UH UK; 3 Medical Physics and Clinical Engineering, Nottingham University Hospitals NHS Trust, Nottingham, NH7 2UH UK

**Keywords:** Multimodality imaging, Hybrid imaging, Intraoperative imaging, Radioguided surgery

## Abstract

**Purpose:**

This review aims to summarise the hybrid modality radioguidance techniques currently in clinical use and development, and to discuss possible future avenues of research. Due to the novelty of these approaches, evidence of their clinical relevance does not yet exist. The purpose of this review is to inform nuclear medicine practitioners of current cutting edge research in radioguided surgery which may enter standard clinical practice within the next 5–10 years. Hybrid imaging is of growing importance to nuclear medicine diagnostics, but it is only with recent advances in technology that hybrid modalities are being investigated for use during radioguided surgery. These modalities aim to overcome some of the difficulties of surgical imaging while maintaining many benefits, or providing entirely new information unavailable to surgeons with traditional radioguidance.

**Methods:**

A literature review was carried out using online reference databases (Scopus, PubMed). Review articles obtained using this technique were citation mined to obtain further references.

**Results:**

In total, 2367 papers were returned, with 425 suitable for further assessment. 60 papers directly related to hybrid intraoperative imaging in radioguided surgery are reported on. Of these papers, 25 described the clinical use of hybrid imaging, 22 described the development of new hybrid probes and tracers, and 13 described the development of hybrid technologies for future clinical use. Hybrid gamma–NIR fluorescence was found to be the most common clinical technique, with 35 papers associated with these modalities. Other hybrid combinations include gamma–bright field imaging, gamma–ultrasound imaging, gamma–β imaging and β–OCT imaging. The combination of preoperative and intraoperative images is also discussed.

**Conclusion:**

Hybrid imaging offers new possibilities for assisting clinicians and surgeons in localising the site of uptake in procedures such as in sentinel node detection.

## Introduction

Radioguided surgery—the intraoperative detection of the emissions from a radioactive tracer—was first used in 1949 for the location of brain tumours during surgery [1]. Since 1949, radioguided surgery has been used to detect a wide variety of tumour types including examples of gastrointestinal, head and neck, gynaecologic, and urologic malignancies [1]. Radioguided sentinel lymph node biopsy (SLNB)—a technique for cancer staging through the investigation of the first lymph node which would be reached by metastasising cells—was first used in 1993 for breast cancer [2], where its use has now become routine [3]. Radioguided SLNB is also in use, or under investigation for use in, melanoma, vulvar, penile, thyroid, colorectal, gastric, head and neck and oesophageal cancers [4].

The majority of radioguided surgical procedures are undertaken with a non-imaging gamma probe, an instrument sensitive to gamma radiation, which produces a numeric and/or audible indication of the magnitude of activity within its field of view. The majority of radioguided procedures (SLNB being the most common) use ^99m^Tc-labelled tracers, which produce gamma radiation with an energy of 140.5 keV. Radionuclides of iodine are also in common use, along with a number of other gamma-emitting radioisotopes [1]. Positron-emitting isotopes such as ^18^F have also been used, with probes either detecting the positron radiation directly [1], the 511 keV gamma photons produced by a positron–electron annihilation [5–7] or optical Cerenkov radiation produced by the decelerating positron [8]. Imaging systems for radioguided surgery—which can provide additional information on spatial distribution—have been developed by a number of researchers and manufacturers. A number of previous reviews [9–11] have detailed these systems, which have a growing user base.

Medical nuclear imaging is a functional imaging technique, where there is not always a direct and intuitive link between features seen in a nuclear medicine image and anatomical landmarks within the body. One solution to assist in interpretation of image information is multimodal, or hybrid, imaging—the acquisition of multiple imaging modalities—which has become common practice. Functional PET images, for example, are now invariably taken in combination with an anatomical X-ray CT [12]. There is now also interest in bringing hybrid modalities to radioguided surgery.

In this review, we discuss intraoperative hybrid techniques currently in use or development and suggest possible directions of future research. Many of these techniques are still in the early stages of technology readiness, with the most advanced technique only entering clinical testing within the last 6 years. Due to this, much of the research summarised here describes translational research either in early clinical pilots or with relevance to upcoming applications in human subjects.

## Method

A literature search was carried out using Scopus and PubMed. References for articles matching the search terms in their abstract, title or keywords were downloaded, with duplicated articles and abstract-only conference proceedings removed. Title and abstract screening was then used to exclude irrelevant articles and to organise the remaining articles into appropriate topics as discussed below. The search included all articles available in these databases up to January 2017. Papers not available in English were excluded. Review papers are included in article counts, but have not been used for analysis unless stated otherwise. Where appropriate, references from papers retrieved in this way were also included for review.

An intentionally broad literature survey was carried out for this topic to ensure that no information was missed due to lack of prior knowledge of the authors. The search terms used are given in Table 1. Eighteen searches in total were carried out to allow all combinations of one term each from column A, column B and column C, with the logical operator ‘AND’ used between terms.

**Table 1:** Search terms used in the survey of hybrid modalities in radioguided surgery

A	B	C
Hybrid	Gamma	Intraoperative
Multimodal	Radio*	Portable
Dual		SFOV

*a standard Boolean search operator indicating a truncation (wildcard) search

This search yielded a total of 2367 retrieved papers, of which 516 duplicate articles and abstract only conference proceedings were removed. The remaining papers were separated into categories as follows (Table 2), with 1426 papers excluded due to not matching with any of the criteria listed. Each category was broken down into a number of subheadings for ease of assessment. Some articles could have been placed in multiple categories—the authors used their judgement to place the article in the single best matched category.

**Table 2:** Outline of criteria for each category used in the hybrid modality review

Category	Criteria	Number of articles
Intraoperative modalities	Intraoperative and/or SFOV imaging with a single modality	250
Preoperative imaging for navigation	Preoperative imaging combined with intraoperative radioguided surgery EXCLUDED: cases with no combination or fusion of modalities, i.e. a preoperative SPECT/CT used to plan radioguided surgery with a non-imaging probe would not be included, but a preoperative SPECT/CT image combined with intraoperative imaging for navigation would be included	9
Hybrid probe development	The development or preclinical testing of hybrid probes/tracers for intraoperative imaging EXCLUDED: probes with insufficiently different modalities (i.e. gamma radiation at two energies) EXCLUDED: theranostic probes where the radio component is not intended for radioguided surgery EXCLUDED: hybrid probes designed for preclinical assessment purposes only	98
Clinical use of hybrid modalities	Clinical use of hybrid modalities EXCLUDED: the ‘hybrid’ use of a visible dye alongside a radioisotope in SLNB	32
Hybrid technologies	Examples of technologies for hybrid intraoperative imaging including developmental and phantom studies	36

## Results

Hybrid systems combining different imaging modalities can provide additional diagnostic information to clinicians and improve patient care. Both PET–CT and SPECT–CT are routinely used and new hybrid systems, such as PET–MRI, continue to emerge. In all these examples, nuclear techniques—which provide functional information—have been combined with techniques that provide detailed anatomical information. Hybrid modalities may also be used to provide complementary information—X-ray mammography may be combined with ultrasound, for example, with the first modality used to identify lesions and the second to characterise them more fully.

Radioguided surgery is a functional technique, with non-hybrid imaging systems only providing an indication of where a source of radioactivity is located within the camera’s field of view, with no relation to anatomical landmarks. Combining these with an anatomical technique such as bright field imaging, ultrasound, MRI or CT might therefore be expected to provide benefits.

Table 3 outlines the hybrid intraoperative imaging types found during the review process with a radioguided component and how retrieved papers were split over developmental stages.

**Table 3:** Review findings for hybrid modalities in radioguided surgery using criteria outlined in Table 2

Category	Number of articles			Total number of articles
	Clinical use	Probe development	Hybrid technologies	
Gamma–bright field	7	2	3	12
Gamma–NIR fluorescence	15	17	3	35
Gamma–ultrasound	1	–	2	3
Gamma–β	1	–	2	3
β–OCT	1	–	1	2
Gamma–MR	–	3	–	3
Pre-/perioperative fusion	–	–	2	2
Total	25	22	13	60

Note that not all modalities would necessarily require a specific hybrid tracer. Reviews covering multiple modalities have not been included here

*OCT* optical coherence tomography

### Gamma–bright field imaging

#### Background and rationale

The use of intraoperative gamma cameras also referred to as portable or small field of view (SFOV) gamma cameras- have been shown to improve localisation for sentinel lymph node biopsy when used with non-imaging gamma probes, particularly for nodes close to high-activity injection sites which cannot be resolved by probes [13]. When gamma imaging is used in isolation, areas of radioactivity are typically shown as bright spots on a black field with no method to directly relate their locations to the surgical field. Attempts to relate the gamma image to the anatomical position on the patient include the use of a laser guide to show the centre of the gamma FOV on the patient, or imaging directly against a patient and then drawing around the gamma camera to mark its position.

For other intraoperative techniques, such as near-infrared fluorescence imaging, functional images are combined with anatomical information from a camera imaging visible light (known as visible, white light or bright field imaging). Fused images combining both modalities have been shown to aid in the localisation of lesions of interest [14]. Applying a similar approach to gamma imaging could allow clinicians to more easily link regions of high activity to locations in the surgical field.

#### Current status

The Freehand SPECT system using a ‘virtual image frame’ has been tested in SLNB for melanoma [15], breast [16] and head and neck [17] cancers and for parathyroid adenomas [18]. This system combines a standard non-imaging gamma probe or portable gamma camera with attachments on both the probe and the patient for detection by an infrared optical tracking system. As the probe is moved around the surgical field, its position and signal are recorded and used to build up a three-dimensional image of areas of radioactivity. An optical camera at a fixed position records a video of the surgical field, and the 3D SPECT image is transformed based on its position and overlaid on the optical image—although generation of the SPECT image requires an approximately 90 s rolling window [19]. This system showed improved detection of sentinel nodes compared to non-imaging gamma probes [17] and the direction and depth estimation provided were found useful by surgeons [20] with the overlay visualisation on the live video improving surgical accuracy [18]. There are, however, some limitations to the system, with the quality of the produced images varying between users [20] and the need for the operating field to remain static over a period of time to ensure accurate localisation [18].

**Fig. 1:**
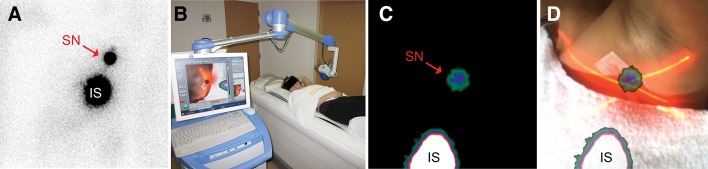
Comparison of preoperative images. In a patient with breast cancer, oblique planar lymphoscintigraphy (**a**) 2 h post-injection showed one sentinel node (SN) in the left axilla. The portable hybrid camera was placed above the lymphatic field to obtain an overview image at a distance of approximately 15 cm (**b**). Standard portable gamma camera imaging showed the injection site (IS) and SN in the same relation to each other, but without any anatomical references (**c**). Combined optical and γ-imaging visualised the image field of view and anatomical SN location in the left axilla (**d**). In the fused image, the SN is visualised on *top* of the *red* laser pointer cross indicating an accurate image fusion (Figure reproduced with permission from [21]—Hellingman et al. A new portable hybrid camera for fused optical and scintigraphic imaging: first clinical experiences. Clinical Nuclear Medicine (http://journals.lww.com/nuclearmed), 41(1):e39–e43, 2016. Promotional and commercial use of the material in print, digital or mobile device format is prohibited without the permission from the publisher Wolters Kluwer. Please contact healthpermissions@wolterskluwer.com for further information)

A portable gamma camera, adapted for hybrid gamma–optical imaging, has been tested intraoperatively. This system was a prototype consisting of the commercially available Sentinella portable gamma camera and an optical module with two optical cameras [21]. The system was calibrated through imaging 15 point sources at a distance of 15 cm and calculating the homography transformation for the modalities. The dual-optical cameras were used to estimate the contours of the surface being imaged. This, along with the calibrated transformation, allowed an optical image to be mapped onto the gamma image—producing a fused image showing both modalities—see Fig. 1. Imaging transformation and fusion required 1–2 s processing time. The average error in location between the gamma and optical images (co-registration errors) was 1 cm at a 15 cm imaging distance, and fusion was not possible at all for imaging distances <5 cm. Fused images were found to be easier to interpret than gamma images alone. Although the co-registration errors meant that they could not be used to directly localise sources, it was found that fused images were particularly useful for determining the orientation and location of the gamma imaging FOV. A similar principle of stereo cameras combined with a portable gamma camera has been used to map gamma images onto an optical image (as opposed to the reverse for the Sentinella system) with smaller co-registration errors when tested in phantoms [22].

With mechanical alignment of the optical and gamma modalities, co-registration errors due to imaging distance and camera alignment can be reduced or eliminated. In the Hybrid Gamma Camera, a mirror is placed in front of the pinhole collimator of a portable gamma camera at 45° to the collimator’s surface, reflecting optical photons to a separate camera [23]. This design means that the FOVs of the optical and gamma components are matched for any imaging distance. The Hybrid Gamma Camera has been used in clinical thyroid imaging [24], but has not yet been tested intraoperatively.

The use of hybrid radioisotope–visible fluorescence probes is common in preclinical imaging and some of these have been translated to the clinic, i.e. for preoperative PET imaging prior to intraoperative optical guidance [25]. In this review, no instances of the hybrid intraoperative use of gamma–visible fluorescence were found. This is likely due to very poor penetration of visible photons through tissue, which would limit visible fluorescence imaging to a surface-only technique. Near-infrared fluorescence is more penetrative than visible light, and hybrid imaging at these wavelengths is discussed in the following section A comprehensive review of hybrid gamma–optical tracers can be found elsewhere [25].

### Gamma–NIR fluorescence imaging

#### Background and rationale

Near-infrared (NIR) fluorescence imaging utilises a tracer that produces optical photons when excited by an external light source. The vast majority of optical light will be absorbed or scattered within the tissue, meaning that optical imaging at depth is limited to a few windows where the major absorption peaks for water, haemoglobin and deoxyhaemoglobin are avoided and therefore transmission is highest (wavelengths of 650–900, 1100–1350, 1600–1870 nm) [26]. Currently, indocyanine-green (ICG) (peak emission at 820 nm) is the fluorophore considered to have the most suitable properties (high photon yield, good tissue penetration and low autofluorescence) that is approved for use in humans (US Food and Drug Administration, European Medicines Agency) [27]; however, the maximum imaging depths are still limited to 5–15 mm [28]. Despite limitations in tissue penetration, NIR fluorescence imaging offers high photon count rates with a high spatial and temporal resolution [29].

The combination of gamma and NIR fluorescence is complementary. Gamma radiation has a very high tissue penetration, allowing detection of sources at depth within the body. However, the high energy of gamma photons means that spatial resolution can be a few centimetres in practice; although this could be improved significantly in theory, any improvement is likely to degrade sensitivity—a more important parameter for intraoperative imaging. The high energy of gamma photons also results in relatively low sensitivity, so images may need to be acquired over several minutes to build up a statistically significant number of photon counts.

Hybrid gamma–NIR fluorescence imaging combines the depth penetration of gamma imaging with the high-quality real-time imaging available for NIR fluorescence. A more complete description of this hybrid modality can be found elsewhere [30].

#### Clinical use

The clinical use of hybrid gamma–NIR fluorescence imaging has so far been limited to the use of ICG as the fluorescence component due to its licence status and availability.

Clinical studies have been particularly focussed on the use of ICG and ^99m^Tc for sentinel lymph node biopsy. The current best practice for SLNB is to inject a ^99m^Tc-based tracer (such as ^99m^Tc-nanocolloid) into the area surrounding the tumour to locate the first-draining ‘sentinel’ nodes during radioguided surgery using a non-imaging probe. Prior to surgery, a blue dye is also injected to visually aid the surgeon in locating the sentinel nodes. The exact procedures vary, depending on the institution, the cancer type and location (i.e. the amount of and type of radiopharmaceutical injected, the typical time between injection and surgery and the use of preoperative imaging all vary). The disadvantages of this technique include difficulty in locating sentinel nodes with a gamma probe and the chance of the blue dye causing ‘tattooing’ of the skin or allergic reactions.

Fifteen clinical investigations of dual localisation with both an ICG and a ^99m^Tc probe for SLNB have been reported in the literature. Of these, all bar two studies used the self-assembling hybrid tracer ICG–^99m^Tc-nanocolloid, with the remaining studies introducing the two tracers separately. Table 4 contains brief details for each of these studies. It is worth noting that, as would be expected for new techniques, the vast majority of these studies have been conducted at, or in collaboration with, a specific institution and using a specific set of instruments. Multicentre studies are necessary to ensure that these results can be replicated by less experienced teams and using differing instrumentation.

**Table 4:** Clinical studies using hybrid gamma–NIR guidance with ^99m^Tc and ICG for SLNB ordered by year of publication

Cancer types	Intraoperative detection	Study pop.	Study location	Year	Refs
Pelvic Prostate carcinoma	G N: Europrobe^a^ G I: Sentinella^b^ (ex vivo) NIR I: Fluorescence laparoscope^c^	11	Dutch Cancer Institute–Antoni van Leeuwenhoek Hospital, Amsterdam, Netherlands	2011	[33]
H&N Squamous cell carcinoma	G N: Neoprobe^d^ G I: Sentinella^b^ NIR I: Photodynamic Eye^e^	14	Dutch Cancer Institute–Antoni van Leeuwenhoek Hospital, Amsterdam, Netherlands	2012	[43]
Pelvic Prostate cancer	G N: Neoprobe 2000^a^ NIR I: (*) Laparoscopic system from component parts^c^	26	Paracelsus Medical University of Salzburg, Austria	2012	[32]
H&N Melanoma	G N: Neoprobe^d^ G I: Sentinella^b^ NIR I: Photodynamic Eye^e^	11	Dutch Cancer Institute–Antoni van Leeuwenhoek Hospital, Amsterdam, Netherlands	2012	[40]
Breast	G N: Europrobe^f^ NIR I: Mini-FLARE^g^	32	Leiden University Medical Centre, Netherlands	2013	[31]
Inguinal Vulvar cancer	G N: Neoprobe^d^ G I: Sentinella^b^ NIR I: Photodynamic Eye^e^	15	Dutch Cancer Institute–Antoni van Leeuwenhoek Hospital, Amsterdam, Netherlands	2013	[34]
Misc. Penile carcinoma, oral cavity tumours, melanoma	G N: Neoprobe^d^ G I: Sentinella^b^ NIR I: Photodynamic Eye^e^	20	Dutch Cancer Institute–Antoni van Leeuwenhoek Hospital, Amsterdam, Netherlands	2013	[36]
Inguinal Penile squamous cell carcinoma	G N: Neoprobe^d^ G I: Sentinella^b^ NIR I: Photodynamic Eye^e^	65	Dutch Cancer Institute–Antoni van Leeuwenhoek Hospital, Amsterdam, Netherlands	2014	[37]
H&N Melanoma, oral cavity squamous cell carcinoma	G N: Neoprobe^d^ G I: Sentinella^b^ NIR I: Photodynamic Eye^e^	25	Dutch Cancer Institute–Antoni van Leeuwenhoek Hospital, Amsterdam, Netherlands	2014	[44]
H&N Melanoma, squamous cell carcinoma, Merkel cell carcinoma, sweat gland carcinoma	G N: Unknown NIR I: (*) Fluobeam^h^ and modified light^i^	40	University Medical Center Klinik für Dermatologie, Essen, Germany	2015	[41]
Inguinal Vulvar cancer	G N: Unknown NIR I: Mini-FLARE^g^	12	Leiden University Medical Centre, Netherlands	2015	[35]
Misc. Melanoma (H&N, trunk, extremities)	G N: Neoprobe^d^ G I: Sentinella^b^ NIR I: Photodynamic Eye^e^	104	Dutch Cancer Institute–Antoni van Leeuwenhoek Hospital, Amsterdam, Netherlands	2015	[42]
H&N Oral cavity squamous cell carcinoma	G N: Neo2000^j^ NIR I: Photodynamic Eye^e^	16	Ehime University Hospital, Japan	2015	[45]
Misc. H&N, penile	G & NIR N: (*) Opto-nuclear probe prototype^f^	9 (in vivo)	Dutch Cancer Institute–Antoni van Leeuwenhoek Hospital, Amsterdam, Netherlands	2015	[38]
Inguinal Penile cancer	G N & NIR I: (*) Europrobe 2^f^ and VITOM^c^ G I & NIR I: (*) Sentinella^b^ and VITOM^c^	11	Dutch Cancer Institute–Antoni van Leeuwenhoek Hospital, Amsterdam, Netherlands	2016	[39]
Misc. Penile, oral squamous cell carcinoma, melanoma (H&N, trunk, extremities)	G N: Unknown G I: Unknown NIR I: (*) Modified and commercial Photodynamic eye^e^	27	Dutch Cancer Institute–Antoni van Leeuwenhoek Hospital, Amsterdam, Netherlands		[50]

Intraoperative detection of gamma photons (G) has been separated into imaging (I) and non-imaging (N). Detection devices used are referenced as footnotes. A (*) indicates that a device that is not commercially available was used in the study. When study location was not explicitly stated, that of the corresponding author has been used

^a^Europrobe, London, UK

^b^Oncovision, Valencia, Spain

^c^Karl Storz, Tuttlingen, Germany

^d^Johnson & Johnson Medical, Germany

^e^Hamamatsu Photonics, Hamamatsu, Japan

^f^Eurorad S.A, Eckbolsheim, France

^g^Curadel, Marlborough, USA

^h^Fluoptics, Grenoble, France

^i^LED DayLite Twin Beam, Designs for Vision, New York, USA, modified with a low pass filter

^j^Neoprobe Corporation, USA

**Fig. 2:**
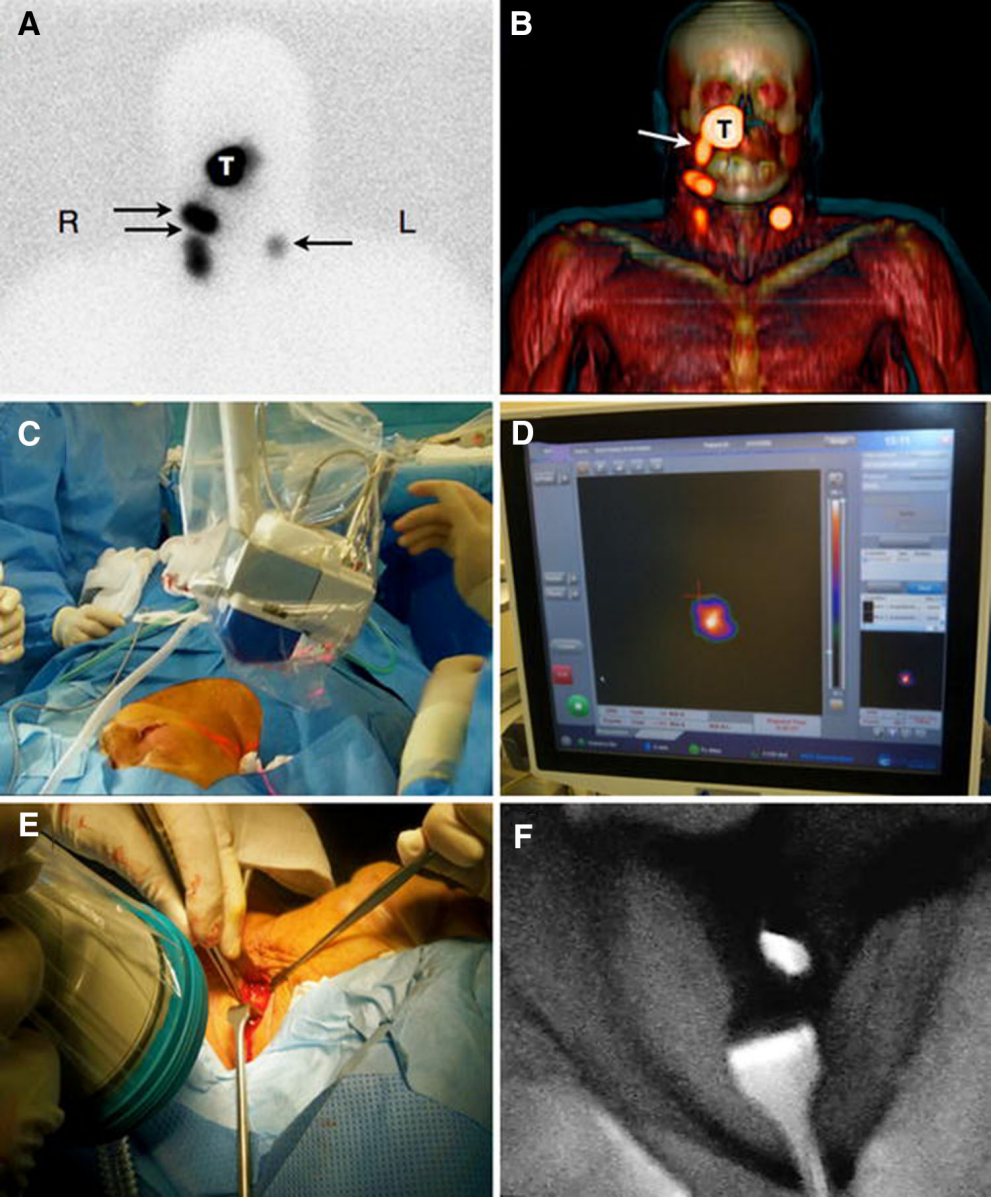
Combined preoperative lymphatic mapping and intraoperative radio- and fluorescence-guided sentinel node biopsy. **a** Early static anterior preoperative lymphoscintigram 10 min after infraorbital peritumoral injection of ICG–99mTc-nanocolloid showing the injection site (T) with lymphatic drainage to two sentinel nodes in the neck on the* right* (R) side and a third one on the* left* (L) side (*arrows*). **b** 3D SPECT/CT image 2 h post-injection providing additional anatomical information with visualisation of a lymphatic duct (*arrow*) originating from the injection site (T). **c**, **d** Intraoperatively, the radioactive component of the hybrid tracer in the left sentinel node is visualised using a portable gamma camera, and its laser pointer guides placement of the incision. **e**, **f** A near-infrared fluorescence camera is used to visualise the fluorescent component of the hybrid tracer in the same (*non-blue*) sentinel node (Figure reproduced with permission from [40])

Hybrid gamma–NIR fluorescence intraoperative guidance has been used for breast [31], prostate [32, 33], vulvar [34, 35] and penile [36–39] cancers along with a range of melanomas [36, 40–42] and cancers of the head and neck (H&N) [36, 38, 40–45]. Figure 2 provides an example series of images from a head and neck procedure.

The greatest amount of research has been conducted for head and neck SLNB with half of the tabulated studies including at least some H&N SLNBs, followed by inguinal SLNB (vulvar and penile) which were included in 6 of the 15 studies. From our review, only a single study investigated the use of hybrid tracing for breast cancer or for non-H&N melanoma, despite these being the most common SLNB procedures. Although this may partly be associated with patient demographics in the investigating research groups, this is also indicative of SLNB procedures with the largest scope for improvement.

Breast SLNB has a reported sensitivity of approximately 100% [46], whereas that of inguinal SLNB for vulvar cancer has been found to be 92% (note that this is a pooled meta-analysis result from 47 papers, including two using the ICG–^99m^Tc technique described here) [47], with an identical sensitivity for penile cancer when the current gold standard blue dye and radiotracer technique is used [48]. The sensitivity of H&N SLNB can vary widely depending on tumour location, ranging from 79 to 87.5% [49]. The lower sensitivity of H&N SLNB is attributed to the complicated structures and drainage patterns within the head and neck, and to the greater likelihood of a node being positioned close to the injection site and so being undetectable with a gamma probe. The poorer sensitivity in these regions may suggest that an improvement on the existing gold standard technique for SLNB is required, and hybrid gamma–NIR fluorescence probes are one possible solution.

All the studies in Table 4 found some utility in the hybrid approach. Nine papers described nodes detectable with NIR imaging but not with the gamma probe [33, 36, 37, 39, 40, 42–45], and seven described nodes detectable with the gamma probe but not NIR imaging [33, 34, 36, 37, 39, 40, 43] (six studies describe both situations [33, 36, 37, 39, 40, 43]). A number of authors stated that the blue dye typically used for SLNB did not provide additional benefits for detection over the dual use of ^99m^Tc and ICG [31, 34, 35, 37, 40, 42].

For detection with gamma probes, difficulties occurred when nodes were relatively close to the tumour/injection site (although in one case this was thought to be due to radioactive decay reducing the signal to undetectable levels [37]). Interestingly, two studies which also used a gamma camera stated that this could be used to detect nodes in regions where background noise swamped gamma probe detection [36, 44].

Near-infrared detection failed when nodes were shielded by tissue; three studies indicated that NIR fluorescence imaging could not detect any nodes prior to incision [39, 41, 45] and two identified nodes that were not detectable in vivo with NIR fluorescence, but were found to be fluorescent when imaged ex vivo [34, 36]. In some cases, the depth limitations for NIR fluorescence imaging were used to estimate node depth and therefore make decisions about the level of invasiveness that would be required to remove these nodes [43, 44].

In addition to benefits seen in detection, some studies noted that logistics could be improved through use of a single injection for both preoperative and intraoperative imaging [27, 37, 40, 42], as binding to the ^99m^Tc-nanocolloid prevented the ICG from being quickly washed out—a known limitation of smaller particles such as the blue dye. A single injectable also ensures registration between the different modalities [34] and optimises logistics [31, 41, 43]. The fact that ICG, along with other NIR fluorescent dyes, is invisible to the human eye means that it does not interfere with the surgical field [43], whereas the blue dye may stain and interfere with primary tumour margin visibility.

The production of the hybrid tracer and so costs per procedure were considered minimal by a number of authors (e.g. [31]). The setup costs in terms of purchasing an appropriate NIR imaging system may be a limitation, although prices vary depending on the systems chosen with some authors considering NIR imaging systems to be of low cost [31] and some of moderate to high cost [42].

One downside of NIR imaging for open surgery is the need to dim the level of surgical lights during imaging to reduce background noise [37, 40, 42, 43] (this is not necessary for laparoscopic surgery). NIR imaging is conducted with the surgical lights dimmed and forceps used to indicate a located node before the lights are turned back on and surgery can progress [50]. Although not noted as a particular logistical challenge in the tabulated studies, this process can disrupt surgical procedure flow and may limit the uptake of NIR imaging in surgical practice. This, however, is not a problem inherent to the modality and engineering solutions are already in development. One option is to replace traditional surgical lights with ones designed to not interfere with NIR fluorescence imaging [41], although this is likely to increase the setup costs significantly. Another solution currently in development is to pulse the excitation light source in sync with the detector frame rate so that pairs of frames, one with and one without fluorescence excitation, are generated [50]. Image subtraction then generates an NIR fluorescence image, with the effect of background ambient light removed. This is a promising technique, although not yet fully optimised—halogen satellite lamps for example still interfere with NIR imaging and must be turned off or directed away from the surgical field [50].

In addition to its use in SLNB, ICG–^99m^Tc-nanocolloid has recently been investigated for use in radioguided occult lesion localisation (ROLL) [51]. In this study, four patients with confirmed tumour-positive ^18^F-FDG-avid lesions were enrolled. The hybrid tracer was injected centrally into each lesion under ultrasound guidance and SPECT–CT imaging was used to confirm its location. Intraoperatively, the lesions were identified using a non-imaging gamma probe (Neoprobe), a gamma camera (Sentinella) and an NIR fluorescence camera (Photodynamic Eye) and excised. The NIR imaging was particularly useful in providing anatomical information about the location of the lesions, which was not available with the intraoperative gamma images [51]. Further evaluation of this technique is needed to determine whether it benefits patients and clinicians.

#### Probe development

The interest in gamma–NIR fluorescence imaging is indicated by the large number of articles describing new probes in development for intraoperative hybrid imaging. Those dealing with NIR fluorescent hybrid tracers can be split into two categories—non-specific probes, including the self-assembled ICG–^99m^Tc-nanocolloid [52] which has now been clinically tested (see Table 4), and specific probes which are designed to target and bind to particular biomarkers. Table 5 provides brief details of the preclinical studies of hybrid targeted tracers.

**Table 5:** Summary of preclinical studies of targeted hybrid gamma–NIR fluorescent probes identified in the literature survey arranged by year of publication

Target	Fluorescent component	Nuclear component	Year	Refs
HER-2 Breast, ovary, endometrium, bladder, lung, colon, H&N [30]	IRDye 800CW^a^	^64^Cu (β)	2010	[59]
α_v_β_3_ integrin Tumour angiogenesis	CyAL-5.5b	^111^In (γ)	2012	[68]
EpCAM Prostate	IRDye 800CW^l^	^64^Cu (β)	2012	[53]
CD105 (endoglin) Breast, pancreatic, prostate, brain	IRDye 800CW^l^	^89^Zr (β)	2012	[60]
EpCAM Prostate	IRDye 800CW^l^	^64^Cu (β)	2013	[64]
CD206 Sentinel nodes	IRDye 800CW^l^	^68^Ga (β)	2013	[65]
GLP-1R Insulinomas, β-cells	Cy5^b^	^64^Cu (β)	2014	[66]
EphB4 Glioblastoma	Cy5.5^c^	^64^Cu (β)	2014	[61]
PSMA Prostate	IRDye 800CW^l^	^111^In (γ)	2014	[54]
CEA Colorectal	IRDye 800CW^l^	^111^In (γ)	2014	[69]
uPAR Colorectal, breast, pancreatic	ZW800-1	^111^In (γ)	2015	[57]
CAIX Clear cell renal cell carcinoma	IRDye 800CW^l^	^111^In (γ)	2015	[55]
CD206 Sentinel nodes	IRDye 800CW^l^	^68^Ga (β) ^99m^Tc (γ)	2015	[67]
Bacterial infection	Cy5	^111^In (γ)	2015	[63]
α_v_β_3_ integrin Tumour angiogenesis, colon adenocarcinoma	Cy5.5	^99m^Tc (γ)	2016	[70]
CAIX Clear cell renal cell carcinoma	IRDye 800CW^l^	^111^In (γ)	2016	[56]
sst_2_ Neuroendocrine	Cy5	^111^In (γ)	2016	[58]

Note that only articles describing specific probes are included, not those that solely described generic platforms for use with different targets, fluorescent or gamma components or those that compare multiple probes. Nuclear components have been labelled as gamma emitting (γ) or positron emitting (β)

^a^LI-COR Biosciences

^b^Lumiprobe

^c^Amersham Pharmacia Biotech

Hybrid optical–gamma tracers are in development to target prostate [53, 54], renal cell carcinoma [55, 56] and colorectal [57], neuroendocrine [58] and breast [59, 60] cancers in addition to glioblastoma [61, 62] and even bacterial infections [63]. The development of these tracers opens up the possibility of the Guided Hybrid intraOperative Specific Targeting (GHOST) technique [30] for a range of cancers. Along with localisation, one goal of the GHOST technique is to greatly improve tumour resection by allowing surgeons to accurately determine tumour margins—something that can often be difficult using visual guidance. Clear tumour delineation through hybrid imaging would allow resection with appropriate margins, ensuring that neither too much nor too little of the surrounding tissue is removed along with the tumour and that no cancerous material remains.

Targeted probe development is split fairly evenly between the use of β-emitting tracers (used for PET) [53, 59–61, 64–67] and gamma-emitting tracers (used for SPECT) [54–58, 63, 67–70]. Most of these probes have been developed with a view to using the gamma component preoperatively and the NIR fluorescence component intraoperatively, but in principle they could also be used for hybrid intraoperative detection. For tracers with a gamma-emitting component, intraoperative procedures could use a standard gamma probe or camera. Intraoperative imaging of PET tracers is more difficult, but there are systems in development to do so either through direct detection of the β radiation [71] or detection of the gamma radiation generated during β^+^/β^−^ annihilation [72].

None of the tabulated probes used ICG as the fluorescence component despite it being the only NIR fluorescence dye with clinical heritage as, unlike the dyes used in Table 5, it cannot be covalently attached to a targeting scaffold [73]. The majority reported using IRDye 800CW available from LI-COR Biosciences with an absorption peak at 774 nm and an emission peak at 789 nm. This dye has recently been licensed for use in clinical trials (US and Europe) with several trials in humans published since 2015 (e.g. [74]). As yet, a hybrid gamma–CW800 probe has not been tested clinically; however this is likely to be an area of growth as licences are now in place.

#### Technological developments

The vast majority of reported clinical uses of hybrid gamma–NIR fluorescence intraoperative imaging, tabulated in Table 4, were carried out using different imaging systems for the gamma and fluorescence modalities. The use of multiple systems can have some benefits—each instrument can be optimised for its own modality and they may provide more flexibility of use for the institutions that purchase them. However, multiple imaging systems, each generally mounted on its own trolley with its own screen or screens, require a significant amount of space within an operating theatre and may require multiple members of staff to operate. Switching between imaging systems may also cause logistical challenges or extend the time of surgery. When two systems are used concurrently, the angle and field of view of each system will differ and images from each system will not align in an intuitive way.

To date, the only hybrid gamma–NIR fluorescence device to be tested intraoperatively is the prototype opto-nuclear probe [38]. This device works similarly to a standard non-imaging gamma probe, providing an audible indication of signal within the FOV, but can be operated in an additional NIR fluorescence sensing mode. When the NIR fluorescence mode is selected, a laser excitation source integrated into the device is switched on and the fluorescence signal is detected by a photomultiplier tube (PMT) behind a high pass filter to block extraneous light—allowing the probe to be used in ambient lighting conditions. The signal from the PMT is fed into the same acoustic system as the gamma signal when in gamma mode operation.

Although surgeons found the use of the opto-nuclear probe intuitive, the lack of imaging capability was seen as a disadvantage in some cases [38]. Unlike gamma count rates which can be considered directly proportional to source activity, NIR fluorescence could not be used quantitatively as signal rates were influenced by intervening tissue, even in excised lymph nodes. Further studies are needed to determine whether the opto-nuclear probe is superior for sentinel node location when compared with a standard gamma probe, or whether a separate NIR fluorescence camera will still be needed to gain the full benefit of the hybrid modality.

The combination of multiple devices (one gamma, one optical) into a single hybrid navigation system has also been trialled [39]. In this study, an NIR fluorescence camera (VITOM) was connected to either a non-imaging gamma probe (GP) or to an SFOV gamma camera (GC) using custom attachments that aligned the focal point of each modality at the optimum working distance for the VITOM. In general, the VITOM–GP was preferred by surgeons as the separate feedback systems (visual for the NIR fluorescence, acoustic for the gamma probe) could be interpreted simultaneously, whereas the VITOM–GC required surgeons to switch between visual signals from two separate screens—however, it was noted that full integration of the modalities onto a single screen in the future may improve surgical logistics. Although the focal points of the devices were mechanically aligned, this was only the case for a single imaging distance (11 cm), which is not the optimum working distance for either the gamma probe or the gamma camera. Operating at different distances meant that the two modalities were not fully aligned—so it was not always possible to see a node within the FOV of both devices simultaneously.

Although the integration of modalities was shown to be synergistic, this study also demonstrated the extent of hardware developments needed to produce a truly integrated system—matched FOVs at a range of imaging distances, adjustable focus for each imaging distance without interrupting surgical workflow and the need to improve the sensitivity of NIR fluorescence imaging at larger imaging distances through improved camera sensitivity and higher powered excitation light sources [39]. Technologies designed for combined gamma–visible imaging (see “Gamma–bright field imaging”) show that the coalignment of these modalities is achievable (as the principles behind visible and NIR fluorescence detection are identical)—recently, an adaptation to the Hybrid Gamma Camera has been tested to show proof of concept simultaneous co-aligned gamma–NIR fluorescence imaging [75]; however it is not known whether the required sensitivity in NIR fluorescence would be achievable with this system.

### Gamma–ultrasound imaging

#### Background and rationale

During ultrasound imaging, ultrasonic waves (acoustic waves with frequencies higher than around 20 kHz) are generated in contact with the patient. The waves pass through the body and are reflected by the interfaces between tissue types. The reflected waves are detected and processed to produce anatomical images, distinguishing between soft tissue, muscle, fat and fluid. Ultrasound imaging systems are relatively compact and portable with a history of intraoperative use for localisation of lesions [76, 77]. Although echogenic contrast agents do exist for ultrasonic imaging, these are not commonly used, and ultrasound is generally considered safe, cost-effective and reliable.

 Combined gamma–ultrasound imaging would include functional data, from the gamma image, with anatomical data from the ultrasound aiding surgical localisation.

#### Current status

**Fig. 3:**
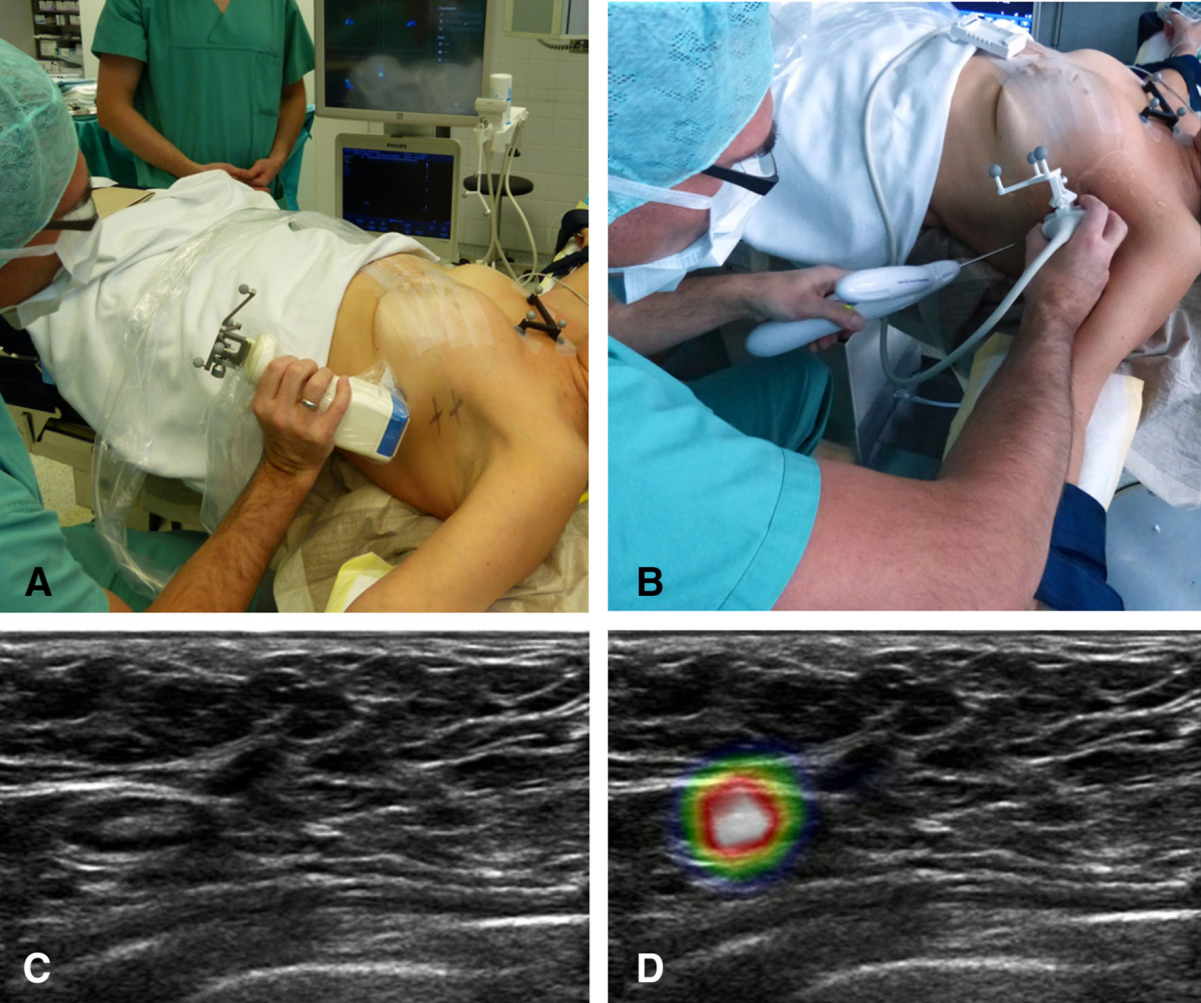
Freehand SPECT–ultrasound in action during sentinel lymph node aspiration biopsy. **a** Freehand SPECT acquisition using a handheld gamma camera as nuclear detector, in a breast cancer patient. **b** Placement of the needle for aspiration biopsy based on freehand SPECT–US image. **c** B-mode image of axilla of patient showing at least one lymph node. **d** Freehand SPECT–US combination highlights the radioactive SLN by making it more prominent (Figure reproduced with permission from [19]—Wendler. Intraoperative 3D nuclear imaging and its hybrid extensions)

Hybrid gamma–ultrasound imaging has been tested intraoperatively using the Freehand SPECT system (described in “Gamma-bright field imaging - Current status”). Among other studies [19], in 2015 the combined gamma–ultrasound system was used to locate sentinel nodes in the head and neck for fine-needle aspiration cytology [78]. 3D gamma images were first generated with the Freehand SPECT system before an ultrasound system attached to the same position-sensing equipment was used. The tracking systems allowed automatic co-registration of the gamma and ultrasound images with deviations limited to a few millimetres; however, this was only the case as specific attention was paid to keeping the patient stationary between imaging procedures to overcome the errors seen in previous studies [78]. The combined imaging procedure identified metastatic lymph nodes which would not have been sampled using ultrasound only guidance; however, sampling errors (intrinsic to aspiration as opposed to full sentinel node excision) did result in false negatives in some cases. This procedure is illustrated for a breast cancer patient in Fig. 3.

A dual gamma–ultrasound imaging device is also under development as part of the ECORAD collaboration, although published work on this to date appears to be limited to Monte Carlo [79] and phantom [80] simulations. These studies provide a proof of principle of the system, but further development is needed to improve camera performance.

### Gamma–β imaging

#### Background and rationale

Some existing radiopharmaceuticals produce both gamma and β radiation (^131^I for example produces 363 keV gamma rays alongside β^+^ emissions) and those that exclusively produce β radiation (such as ^18^F) can be detected via the 511 keV emissions from β^+^/β^−^ annihilations. Although gamma rays can travel many centimetres through tissue, penetration of β radiation is limited to a few millimetres.

For radioguided surgery near organs with a large radiotracer uptake (e.g. the heart, bladder or brain), the penetration of gamma rays can cause background contamination from these organs and hamper detection [71]. Combined gamma–β detection would be complementary, with gamma detection used for general localisation at depth and β detection for high-resolution localisation.

#### Current status

Separate non-imaging gamma and β probes have been tested in concert for a range of cancers (including breast, thyroid, colorectal and gastric)—although the sensitivity of the gamma probe to 511 keV photons was found to be too low to be clinically useful. Phantom studies showed that the amount of tissue needed for localisation with the β probe was an order of magnitude smaller than that needed for detection with a whole body PET. The β probe was considered useful in confirming that no tumour tissue remained in the resection bed. However, the short range of positrons in tissue means that tumour sites covered with benign tissues may potentially go unnoticed—this suggests that combination with a gamma probe of an appropriate sensitivity would be beneficial [81].

Combined systems for both gamma and β detection are in development. A multilayered detector has been shown to discriminate between gamma and β radiation and provide spectroscopic information for both [82]—although this system has not been designed with medical applications specifically in mind. A probe combining both non-imaging gamma detection and β imaging capabilities is in development for intraoperative use [83]. The dual-channel probe has been tested in a variety of phantoms and is able to produce images with a sub-millimetre spatial resolution, detecting simulated tumours with background ratios as high as 10:1. When simulated tumours were placed under chicken skin, variation in skin thickness was seen to have a large effect on the detected signal indicating that, as in NIR fluorescence, variable attenuation prevents β imaging from being a quantitative technique.

### Gamma–Cerenkov luminescence imaging

Cerenkov luminescence is produced when a charged particle moves through a medium at a speed greater than the speed of light in that medium. As the particle travels though the medium, energy is transferred to the medium. After the particle has passed, electrons in the medium relax and, in doing so, emit optical photons—typically in visible or ultraviolet wavelengths [84].

For intraoperative imaging, a β-emitting radiopharmaceutical, such as ^18^F, is administered. The rationale for gamma–Cerenkov luminescence imaging is similar to that for gamma–β imaging. Gamma detection may be used for coarse localisation at depth, while Cerenkov luminescence imaging offers enhanced spatial resolution but with minimal depth penetration.

**Fig. 4:**
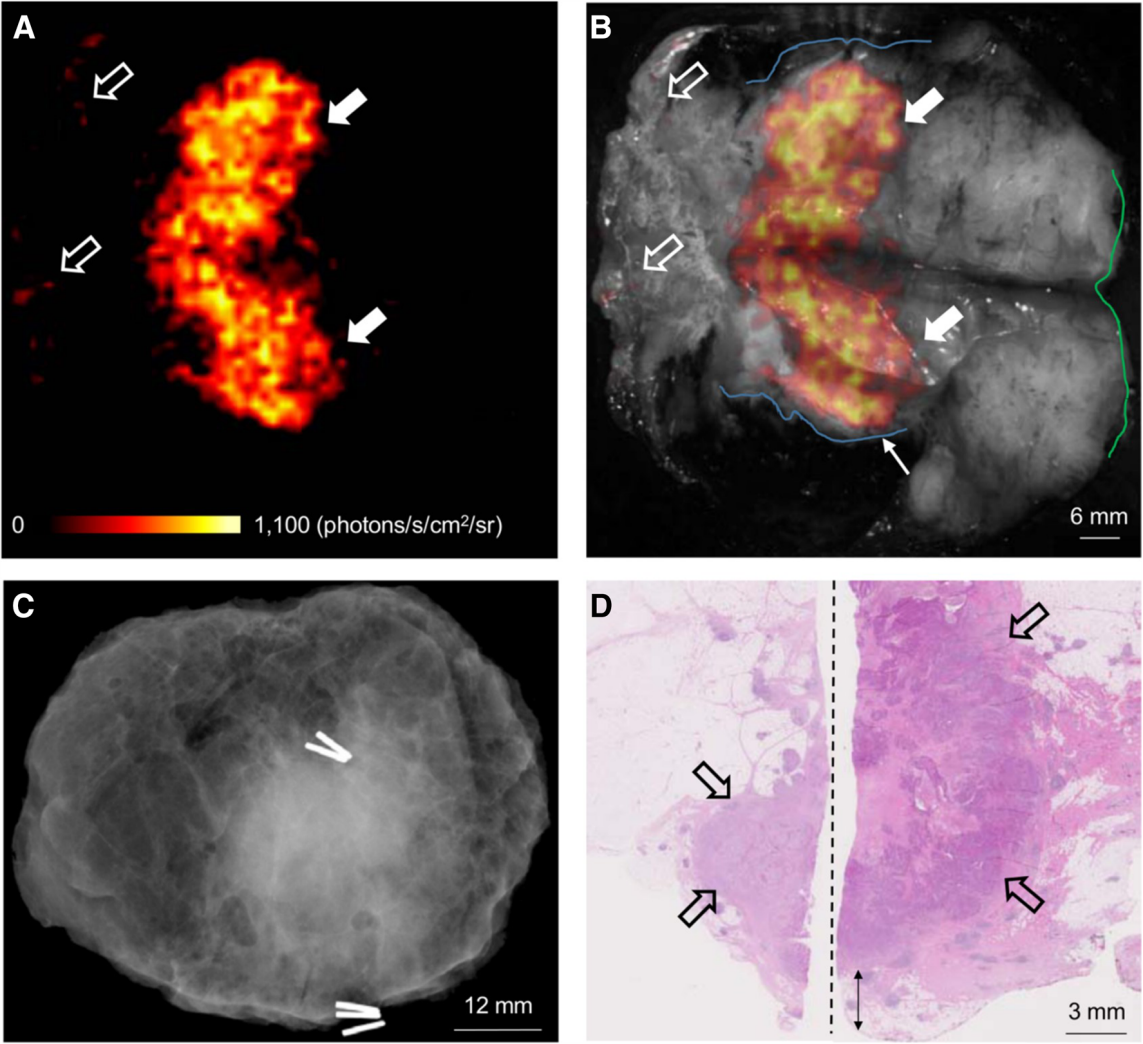
Wide local excision specimen from a patient with a grade 3, ER-/HER2-, no special type (NST) carcinoma. **a** Cerenkov image; **b** Greyscale photographic image overlaid with Cerenkov signal. An increased signal from the tumour is visible (*white arrows*); the mean radiance is 871 ± 131 photons/s/cm^2^/sr and the mean tumour to background ratio is 3.22. Both surgeons measured the posterior margin (outlined in *blue*) as 2 mm (*small arrow*); a cavity shaving would have been performed if the image had been available intraoperatively. The medial margin (outlined in *green*) measured >5 mm by both surgeons. Pathology ink prevented assessing the lateral margin; a phosphorescent signal is visible (*open arrows*). **c** Specimen radiography image. The absence of one surgical clip to mark the anterior margin, and the odd position of the superior margin clip (*white arrow*) prevented reliable margin assessment. **d** Combined histopathology image from two adjacent pathology slides on which the posterior margin (*bottom* of image) and part of the primary tumour are visible (*open arrows*). The distance from the posterior margin measured 3 mm microscopically (*two headed arrow*). The medial margin is >5 mm (not present in image) (Figure reproduced with permission from research originally published in JNM [96] © by the Society of Nuclear Medicine and Molecular Imaging Inc.)

Due to the low intensity of Cerenkov luminescence, it is best suited for endoscopic or ex vivo imaging where an entirely light-tight environment can be ensured (see Fig. 4 for an example of this technique). Cerenkov luminescence imaging has been used clinically in both these situations and has also been used in conjunction with preoperative PET imaging [84]; however, this review found no instance of usage of hybrid intraoperative imaging. Cerenkov luminescence imaging is still a relatively new intraoperative technique, and the development of hybrid systems is likely to follow after optimisation of this technique.

### Β–OCT imaging

#### Background and rationale

Optical coherence tomography (OCT) is an analogous technique to ultrasound imaging where near-infrared light is used in place of ultrasound. Unlike ultrasound imaging, OCT does not need to be conducted in direct contact with the patient. Spatial resolution is of the order of a few micrometres and therefore OCT can be used to investigate tissue microstructure, but imaging can only occur at depths of a few millimetres [Stammes et al. submitted to Ann. Surg.]. OCT is not yet in routine clinical use, but has been used for intraoperative imaging (mainly ex vivo [Stammes et al. submitted to Ann. Surg.] with early in vivo trials [85]) to assess the margins of excised tumours.

Both β detection and OCT are superficial techniques, with β detection providing functional information, and OCT anatomical information (albeit on a very small scale).

#### Current status

**Fig. 5:**
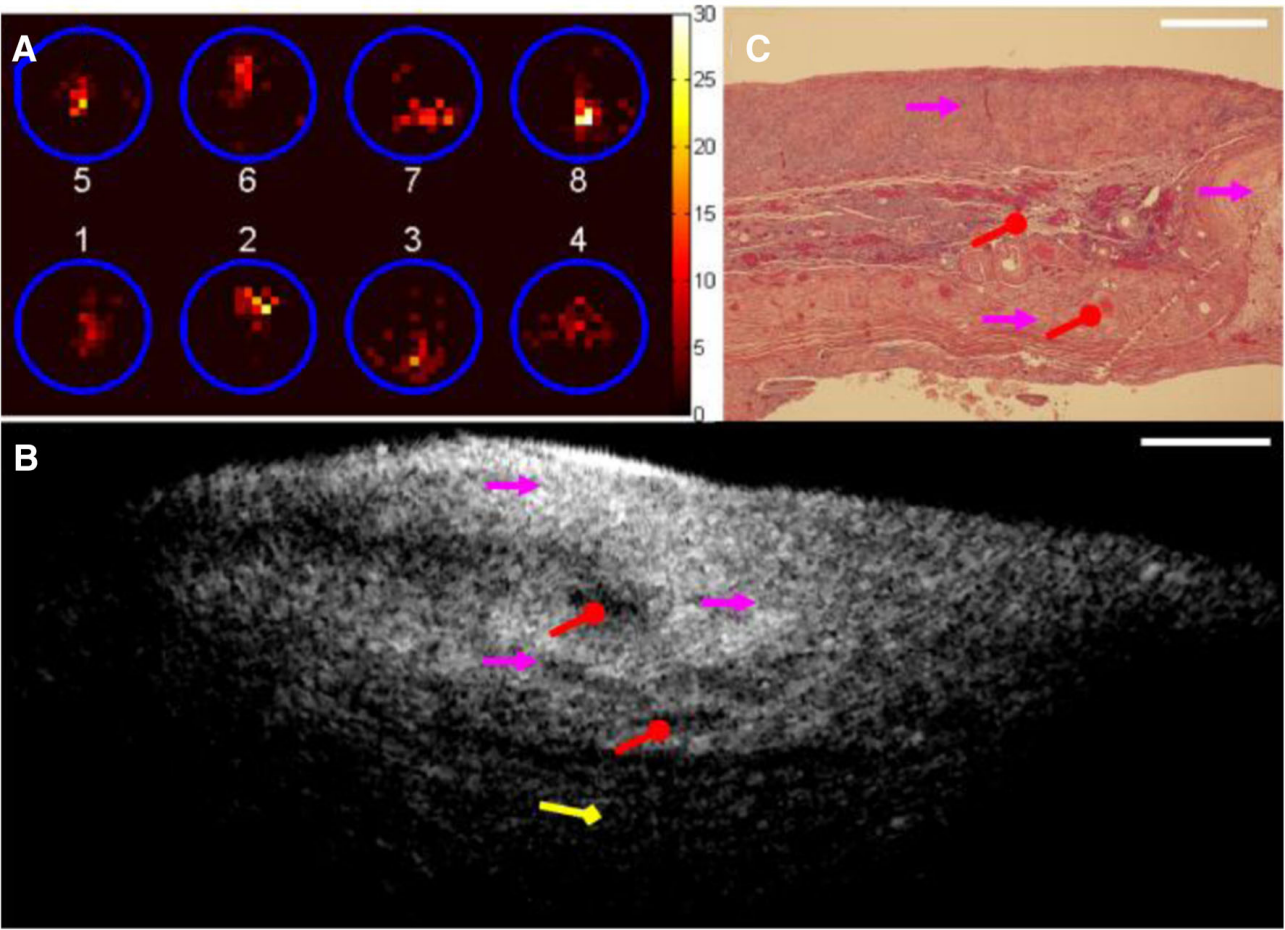
One set of images obtained from the right ovary (abnormal) of a patient. **a** Positron distribution map; **b** one representative OCT image obtained from a sequence of co-registered OCT images; **c** corresponding 40× H&E histology. *Red circle* corpus albicans; *red arrow* collagen; *purple arrow* congested vessels; *yellow arrow* dermoid tumour. The OCT image size is 2 mm (depth) × 5 mm (lateral) (height × width); the histology size is 2 mm × 2.6 mm (height × width); the *white scale bar* is 0.5 mm (Figure reproduced with permission from [87])

A prototype hybrid β–OCT probe currently in development comprises a central scanning OCT fibre, surrounded by scintillating fibre tips able to detect β radiation [86]. The detection of β particles is intended for coarse localisation of activity, with the scanning OCT providing high-resolution co-registered structural imaging of the tissue. This has been tested in ovarian cancer—a cancer that is often diagnosed at late stages due to difficulties in detection. Although PET imaging with ^18^F-FDG can detect ovarian tumours, the low background to tumour signal ratio can make early stage cancers undetectable [87] and so more sensitive diagnostic tools are needed. The hybrid probe has been used for ex vivo ovary imaging after injection of ^18^F-FDG. Positron count rates of 7.5/8.8-fold higher were found between malignant ovaries and abnormal/normal ovaries. OCT imaging of malignant and abnormal ovaries revealed many detailed morphologic features that could be potentially valuable for evaluating local regions with high metabolic activities and detecting early malignant changes of the ovary, with structures seen intraoperatively that were then confirmed in histology (see Fig. 5). Improvements to sensitivity and miniaturisation of the device (to fit a standard laparoscope accessory port) are planned before future in vivo testing [87].

### Gamma–MR imaging

Magnetic resonance imaging (MR) requires that hydrogen nuclei in the body are excited by radiofrequency pulses while an external magnetic field is applied. The rate of spin relaxation differs for atoms within different tissue and these rates can be used to build up an image of tissue distribution. Although some forms of MR imaging are functional, it is typically thought of as an anatomical technique and is particularly suited to imaging soft tissues. The need for very strong magnetic fields has limited the use of MR imaging intraoperatively, with specialist operating rooms and large and expensive equipment required [88].

Although hybrid gamma–MR probes are in development [89–91], these are likely to be best suited to preoperative MR imaging and intraoperative gamma detection rather than for truly hybrid intraoperative imaging. With the advancement of preoperative and intraoperative image fusion (see “Fusion of preoperative and intraoperative images”), these probes may find more surgical utility.

### Fusion of preoperative and intraoperative images

Although not the main focus of this review, the fusion of preoperative and intraoperative images is an adjacent field to intraoperative hybrid imaging. In general, this technique aims to fuse images taken during surgery with those taken prior to surgery. One benefit of preoperative/intraoperative image fusion is the ability to use preoperative imaging techniques including MRI, SPECT, PET–CT, etc. which are not available in the operating theatre and which provide different or improved information such as 3D vs planar, higher sensitivity and the ability to image different structures. Preoperative images such as these are already widely used to inform radioguided surgery [92]; however, it can be difficult for surgeons to directly relate what they see in the surgical field to features in the preoperative image. One way to overcome this could be to fuse preoperative and intraoperative images into an augmented reality display.

The Freehand SPECT tracks the location of a gamma detection device and uses this to build up a 3D image of the distribution of a radioisotope. In “Gamma-bright field imaging - Current status” it was discussed that this could then be overlaid on a video of the surgical field. This system has been adapted to act as an augmented reality navigation system, with the tracked location of the gamma probe used to display the preoperatively acquired images from the point of view of the probe, or to overlay them on the surgical field. This has been tested in phantoms for preoperative MR images [93] and intraoperatively for SPECT–CT images [94]. In principle, intraoperative Freehand SPECT images could in addition be fused with the preoperative images, with both overlaid on the video of the surgical field for augmented reality surgical guidance.

The registration of pre/intraoperative images can be a complicated and mathematically intensive process [95], particularly as for intraoperative use real-time (and preferably entirely automatic) registration would be required. Further difficulties occur when areas of the field are deformed during surgery or if there has been an internal shift in the position of features after preoperative imaging occurs, and so it is unlikely that augmented reality based on preoperative imaging can ever be used in isolation. It may be that combined pre/intraoperative images will prove the ideal solution, but more developmental work is needed to make this a practical possibility.

## Discussion

Hybrid intraoperative radioguided surgery is a broad and steadily growing field. To date, the majority of clinical studies have investigated gamma–NIR fluorescence imaging in SLNB. SLNB biopsy is a well-established, common and reliable technique and can be considered a useful testing ground for newer intraoperative imaging modalities. NIR fluorescence imaging is relatively affordable which may also have encouraged the extent of testing of this technique. The focus of most of these studies is SLNB for cancers of the head and neck—a procedure where optimisation is still needed and where a hybrid approach may offer the most benefit. A number of targeted hybrid gamma–NIR fluorescence probes are in development, and it is expected that the use of intraoperative gamma–NIR fluorescence imaging for tumour resection will be a large area of research in the future.

A range of other hybrid techniques are also in development, with some focussed on providing additional anatomical information to surgeons (gamma–bright field, gamma–ultrasound, β–OCT) and some aimed at complementing traditional radioguided surgery (overcoming known spatial resolution and background contamination concerns as in gamma–NIR fluorescence and gamma–β imaging). None of these techniques is likely to prove a universal solution for all radioguided procedures; however, it is envisaged that with further development all these techniques can be offered as additional tools for surgeons, assisting with the procedures they wish to perform.

A summary of the findings of this review can be given as follows;

1. Hybrid radioguided surgical techniques are designed to combine functional and anatomical information, or to improve the spatial resolution of radioguided techniques while maintaining depth penetration.
2. The goal of hybrid radioguided surgery is to reduce surgical times and/or increase surgical precision when compared with traditional radioguided techniques.
3. NIR fluorescence-radioguided surgery for SLNB is the hybrid technique with the longest heritage of clinical use. Studies have reported this technique to be equally sensitive for SLNB without the use of blue dye. Both gamma and NIR modalities have been reported to detect nodes that are not seen with the other modality.
4. A large number of targeted probes appropriate for NIR fluorescence-radioguided surgery in tumour resection are in development. With recent licensing of the NIR dye CW800 for clinical trials, targeted probes are expected to begin reaching clinical tests in the near future.
5. Each hybrid approach in development has different strengths and weaknesses. The uptake of these techniques will depend on proof of clinical benefits as they enter higher developmental levels.
